# Emergence of the CD226 Axis in Cancer Immunotherapy

**DOI:** 10.3389/fimmu.2022.914406

**Published:** 2022-06-24

**Authors:** Michael Conner, Ken W. Hance, Sapna Yadavilli, James Smothers, Jeremy D. Waight

**Affiliations:** Oncology R&D, GlaxoSmithKline, Collegeville, PA, United States

**Keywords:** cancer immunotherapy, NK cells, T cells, Treg cells, antibodies, fc gamma receptors (FcγR)

## Abstract

In recent years, a set of immune receptors that interact with members of the nectin/nectin-like (necl) family has garnered significant attention as possible points of manipulation in cancer. Central to this axis, CD226, TIGIT, and CD96 represent ligand (CD155)-competitive co-stimulatory/inhibitory receptors, analogous to the CTLA-4/B7/CD28 tripartite. The identification of PVRIG (CD112R) and CD112 has introduced complexity and enabled additional nodes of therapeutic intervention. By virtue of the clinical progression of TIGIT antagonists and emergence of novel CD96- and PVRIG-based approaches, our overall understanding of the ‘CD226 axis’ in cancer immunotherapy is starting to take shape. However, several questions remain regarding the unique characteristics of, and mechanistic interplay between, each receptor-ligand pair. This review provides an overview of the CD226 axis in the context of cancer, with a focus on the status of immunotherapeutic strategies (TIGIT, CD96, and PVRIG) and their underlying biology (i.e., *cis*/*trans* interactions). We also integrate our emerging knowledge of the immune populations involved, key considerations for Fc gamma (γ) receptor biology in therapeutic activity, and a snapshot of the rapidly evolving clinical landscape.

## Introduction

With the widespread clinical application of the ‘first generation’ of immune checkpoint inhibitors (ICI), namely antibody-mediated blockade of cytotoxic T lymphocyte-associated protein-4 (CTLA-4) and programmed cell death protein/ligand-1 (PD-[L]1), immunotherapy has become a mainstay approach for the treatment of cancer (1). However, despite a wealth of evidence supporting the use of anti-CTLA-4 and anti-PD-1/L1, many patients fail to derive meaningful benefit – highlighting the need for alternative and complementary immunotherapeutic interventions (2). In this regard, engagement of novel pathways, cell types, and combinations may provide therapeutic options for patients wherein the pre-existing host and tumor microenvironment factors do not favor current immunotherapeutic agents or where adaptive resistance has occurred (3).

**Figure 1:**
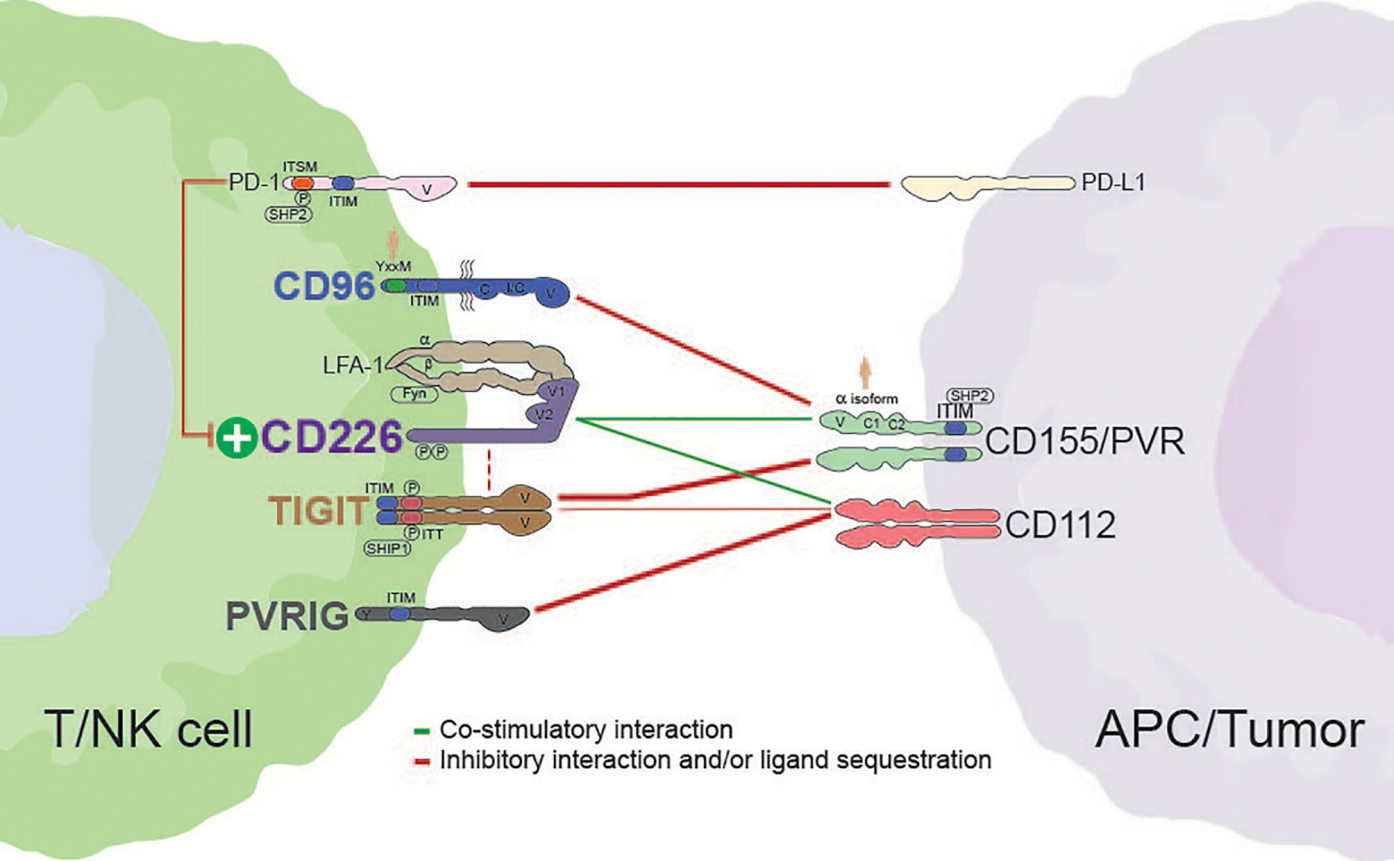
Interactions between members of the CD226 axis. Dashed red lines indicate the potential for *cis* interactions/inhibition. The weight of each line is representative of the relative strength of interaction. Alternative interactions not shown (e.g., CD96 with CD111; TIGIT with CD113 or PVRL4). Human silhouettes signify that a motif or isoform is not present in rodents. APC, antigen presenting cell; V, variable-like domain; C, constant-like domain; I/C, I/C-like folding pattern present in isoform 2 of human CD96; P, tyrosine phosphorylation site; SHP2, Src homology 2-containing phosphotyrosine phosphatase; ITIM, immunoreceptor tyrosine-based inhibition motif; SHIP1, Src homology 2-containing-inositol-phosphatase-1.

For more than a decade, the CD226 axis have been characterized in the context of natural killer (NK) and T cell biology (4). At the core of this family, T cell immunoreceptor with Ig and ITIM domains (TIGIT) and CD96 (TACTILE) effectively compete with CD226 (DNAX Accessory Molecule-1 [DNAM-1] for binding to the necl protein CD155 (poliovirus receptor [PVR). This regulatory network is reminiscent of the interplay between cytotoxic T-lymphocyte-associated protein-4 (CTLA-4)/CD28 and B7 (CD80/CD86), where a common ligand (CD155) is shared between costimulatory (CD226) and co-inhibitory receptors (CD96 and TIGIT) (**Figure 1**) (5). While a parallel with the CTLA-4 axis could provide some mechanistic insight to the CD226 axis, there are some important differences in cellular expression, potential for direct inhibitory receptor signaling, and the potential impact of soluble ligand (6, 7). Adding to this complexity, the recently described PVRIG has been shown to compete with CD226 binding to CD112, another ligand in this axis (**Figure 1**) (8–10).

The contiguous nature of the CD226 axis begs several questions. For example, are the family members redundant such that concurrent antagonism of multiple receptors is necessary to reveal their full functional potential, or are individual receptors dominant under distinct contexts? A thorough understanding of the dynamics of each ligand-receptor pair will be critical for the mechanistic deconvolution of a seemingly redundant family. These relationships may also inform the best approaches for successful therapeutic intervention (i.e., best indications to target individual or multiple receptors, mono- or bi-specific strategies, etc.). To help address this, we explore the structural characteristics, reported interactions, and expression patterns for each immune receptor in the CD226 axis (**Table 1**). We also discuss the potential for cell-intrinsic activity and present the available evidence supporting combinations with antibodies targeting the CD226 axis. In addition, given the importance of Fc-Fc gamma (γ) receptor co-engagement to CTLA-4 antibody function, and inherent similarities with the CTLA-4/B7/CD28 family, we briefly discuss the potential role of FcγRs in promoting the functional activity of antibodies targeting the immune receptors in the CD226 axis (14–16). Finally, we provide a snapshot view of the current therapeutic landscape for the CD226 axis, surveying the available clinical data for each target and highlighting current indications, safety considerations, and combination strategies for each target.

**Table 1:** Expression of CD226 axis members on human immune populations.

	TIGIT	CD96	PVRIG	CD226
**CD4+ T cells: Naïve** ^a^	+/-	+/-	+/-^*^	++
**CD4+ T cells: EM** ^a^	++	+++	+/-^*^	+++
**CD4+ T cells: CM** ^a^	+	++	+/-^*^	+++
**CD4+ T cells: TEMRA** ^a^	+/-	+/-	ND	++
**CD4+ T cells: Treg** ^a^	++++	+/-	+/-^*^	++
**CD4+ T cells: Tfh** ^c^	++	ND	ND	ND
**CD8+ T cells: Naïve** ^a,b^	+/-	+	+/-	++
**CD8+ T cells: EM** ^a,b^	++	+++	+++	+++
**CD8+ T cells: CM** ^a,b^	+/-	++	+	+++
**CD8+ T cells: TEMRA** ^a,b^	+++	+	+++	++
**MAIT cells** ^a^	-	++	+^*^	+++
**γδT cells** ^a^	+++	+/-	+^*^	+++
**B cells** ^a^	+	+/-	-^*^	-
**NK cells** ^a,b^	++	++	++	+++
**NKT cells (CD56+ T cells)** ^a^	++	++	ND	+++
**Myeloid** ^a^	-	-	-^*^	++

Analysis based on human PBMCs. Expression may vary depending on tissue type and indication (e.g., cancer). Symbols (+ and -) represent relative and qualitative expression of each receptor, wherein + is positive expression and – or -/+ is negative or minimal/variegated expression, respectively. ND, not determined. a (11); b (12); c (13); *RNA analysis only (Human Cell Atlas).

## The Core of the Axis: CD226

### Discovery, Structure, and Interactions

By virtue of its role in cytotoxic T cell maturation and platelet activation, CD226 was initially identified as T lineage-specific antigen (TLiSA1) and platelet and T cell antigen 1 (PTA1) shortly thereafter (17, 18). CD226, or DNAX accessory molecule 1 (DNAM-1), has since been thoroughly characterized as a T and NK cell co-stimulatory receptor responsible for orchestrating the signaling of shared ligands CD155 and CD112 (8, 19–22). Analogous to CD28 in the B7/CTLA-4 axis, CD226 has a reduced affinity for shared ligands CD155 and CD112 relative to the inhibitory receptors TIGIT, CD96, and PVRIG, thus creating a layer of immune regulation *via* competitive inhibition (9, 19, 23–26). Exemplifying its important role in immune homeostasis, CD226 genetic polymorphisms are associated with various immune pathologies (27–29). Similar correlations are lacking for TIGIT, CD96, and PVRIG, highlighting the central nature of CD226 in controlling immune activity within the family.

**Figure 2:**
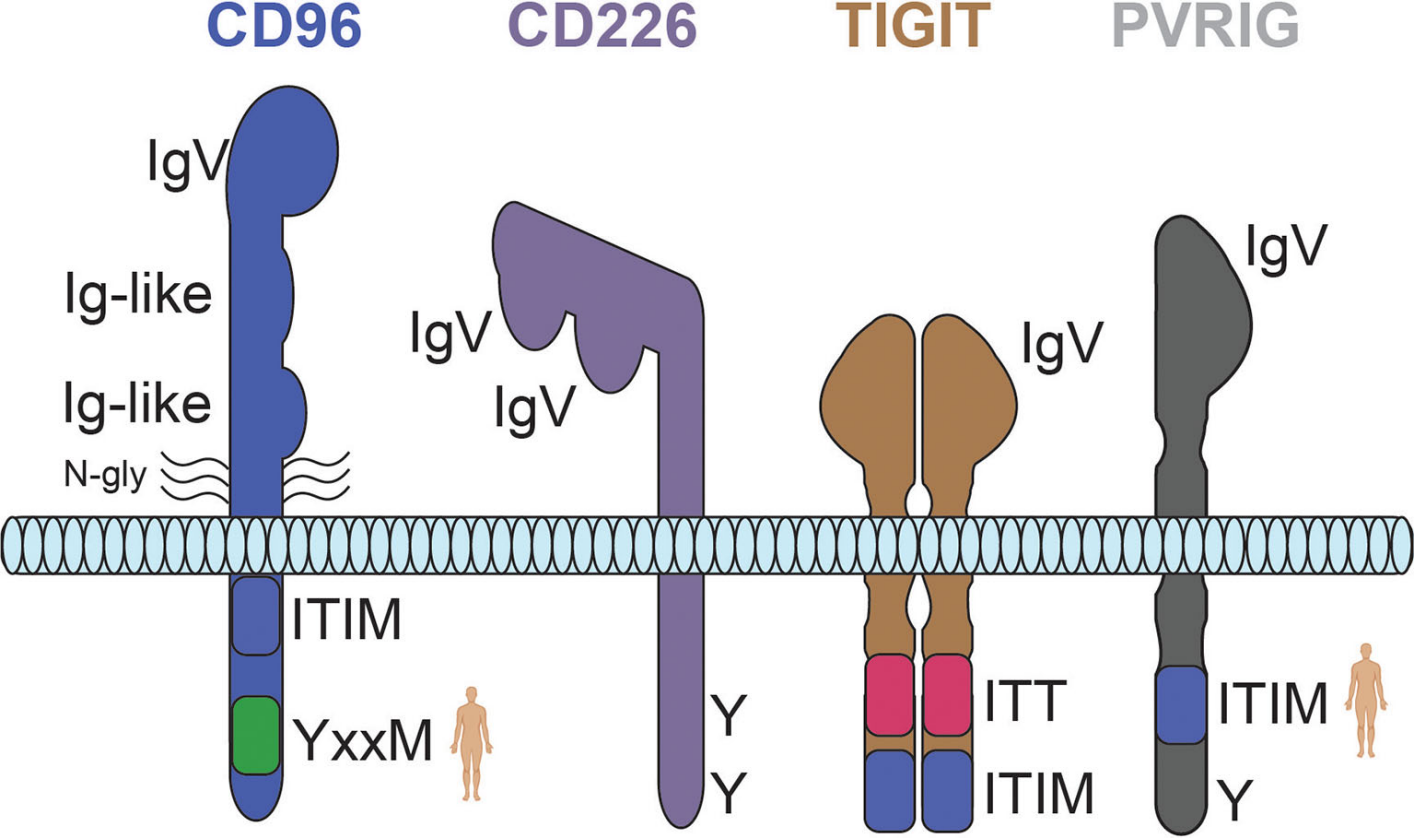
Predicted structures for the CD226 axis receptors CD96, CD226, TIGIT, and PVRIG. The weight of each line is representative of the relative strength of interaction. Human silhouettes signify that a motif is not present in rodents. N-gly, n-linked glycosylation; Y, tyrosine residue; ITT, immunoglobulin tail tyrosine motif; ITIM, immunoreceptor tyrosine-based inhibition motif.

The extracellular region of CD226 forms a unique structure whereby its two IgV domains (domain [D]1 and D2) are linked in a side-by-side arrangement (**Figure 2**). As a result, while interactions are primarily mediated by a conserved ‘lock-and-key’ motif in D1, the second extracellular domain (D2) can also contribute to ligand binding (25, 26). The intracellular region of CD226 harbors a conserved tyrosine (Y)/asparagine (N) motif (D/EIYV/MNY), which engages with multiple proteins, including growth factor receptor bound protein 2 (Grb2) (30). Site-directed mutagenesis of Y319 abrogates CD226-induced cellular cytotoxicity (30) (**Table 2**). This residue (Y319) has also been associated with regulation of CD226 expression *via* Casitas B-lineage lymphoma proto-oncogene-b (Cbl-b)-dependent ubiquitination/degradation following CD155 engagement (37). Additionally, although it appears to be contextual, co-localization with lymphocyte function-associated antigen 1 (LFA-1) during immune synapse formation has also been described (**Figure 1**) (30, 32).

**Table 2:** CD226 axis receptor ICD mutational studies and associated functional effects.

Receptor	System (cell type)	Modifications	Finding(s)	Ref.
CD226 (human)	BW5147 (T cell line, mouse), human CD226 ectopic	S329F mutation; various truncations of intracellular domain	Prevention of protein kinase C (PKC) phosphorylation and subsequent cellular adhesion	(31)
CD226 (human)	Jurkat and NKL (T and NK cell lines, human)	WT in NKL cells. Mutation of S329F in Jurkat cells	S329F mutant failed to associate with LFA-1	(32)
CD226 (human)	Jurkat (T cell line, human) and COS-7 (fibroblast line, monkey)	Y322F	Fyn induced phosphorylation at Y322, abrogated by Y322F mutation.	(32)
CD226 (mouse)	Primary murine NK cells and YTS (human NK cell line)	Ectopic expression of mouse CD226 in YTS cells	Increased phosphorylation of Erk and Akt, as well as calcium flux and target cell lysis following CD226 engagement	(30)
CD226 (mouse)	YTS (NK cell line, human) and transgenic mice	Y319F, S326A	Y319F modification in CD226 attenuated mouse NK cell cytotoxicity and IFNg production	(30)
CD226 (mouse)	Synthetic peptide corresponding to 315-333 of mouse CD226	Synthesized with phosphorylation at Y319, D321Q	Phosphorylation of the amino acid corresponding to Y319 led to capture of Grb2. Mutation of the asparagine at +2 position led to loss of Grb2 binding	(30)
CD226 (human)	Jurkat (T cell line, human) or primary (T cells, human)	Y322A, S329A	Y322A modification, but not S329A, reduced downstream CD226 signaling after incubation with CHO-OKT3-PVR cells	(33)
TIGIT (human)	Jurkat (T cell line, human)	Y225A, Y231A	Y225A/Y231A dual mutation rescued CD69 expression on Jurkat cells following exposure to superantigen and CD155	(33)
TIGIT (human)	YTS (NK cell line, human)	Truncation (Y231stop); Y231A	Rescue of CD155-TIGIT induced inhibition of YTS (NK) mediated cytotoxicity following truncation or Y231A mutation in TIGIT	(10)
TIGIT (human)	YTS (NK cell line, human)	Y225A, Y231A	CD155 induced TIGIT phosphorylation. Pervanadate treatment-induced phosphorylation of TIGIT was prevented with Y225A or Y225A/Y231A, but not Y231A alone	(34)
TIGIT (human)	YTS (NK cell line, human)	Y225A, Y231A	TIGIT Y225 associates with Grb2, leading to downstream inhibitory function. Nominal rescue of cytotoxicity with Y231A mutation	(34)
TIGIT (human)	YTS (NK cell line, human)	Y225A, Y231A	Reduced association with β-arrestin 2 following mutation Y225A or Y225A/Y231A, but not Y231A alone	(35)
TIGIT (human)	Jurkat (T cell line, human)	ICD truncation, Y225F, Y231F	TIGIT intracellular domain is not required to prevent PVR-induced CD226 phosphorylation	(36)
TIGIT (human)	Cell-free liposome	ICD expressed on cell-free liposome	Lack of Shp2, Shp1, Zap70, Grb2, SHIP-1, or P50a recruitment following phosphorylation of TIGIT ICD by Fyn	(36)
CD96	Undescribed	Undescribed	Undescribed	None
PVRIG (human)	HEK293T (kidney cell line, human) and MOLT4 (T cell line, human)	Y233F, Y293F	Y233F mutation reduced phosphorylation after pervanadate treatment. PVRIG associates with SHIP and Shp1/2 following pervanadate treatment	(9)

### Expression and Regulation

CD226 is broadly expressed on innate and adaptive immune populations and, like the associated inhibitory receptors within the axis, can be induced on peripheral human T cells following T cell receptor (TCR) activation (**Table 1**) (11, 38). Notably, the expression of CD226 on activated human T and intratumoral NK cells tracks closely to that of CD96 (11, 39). While co-expression could suggest some level of reactive regulation within the axis, it remains unclear if it is simply correlative or if it is biologically meaningful.

Another mechanism to temper immune activation could be the direct regulation of CD226 expression (i.e., by ligand-induced internalization/endocytosis or cleavage), as evidenced by its modulation in various disease settings including chronic viral infection and cancer (37, 40, 41). For example, in non-small cell lung cancer (NSCLC), CD226 expression is reduced on tumor-infiltrating NK cells relative to cells from normal adjacent tissues (NAT) and peripheral blood (42, 43). Moreover, CD226 has been shown to be sensitive to changes elicited by various therapeutics, such as chemotherapy (44).

While ligand-based interactions have primarily been implicated in driving the loss of cell surface CD226, recent work by Sun et al. also suggests that this effect could be mediated by soluble factors like transforming growth factor beta (TGF-β1) (37, 39, 45, 46). It is unclear if this is a direct or indirect effect, however, TGF-β-dependent modulation of CD226 has the potential to skew axis signaling in the tumor microenvironment (TME), enhancing the potential for immune evasion. Additionally, increased soluble CD226, and related loss of cell-surface expression, has been observed in the sera of cancer patients, suggesting some level of protease-dependent biology (47). Regardless of mechanism, the reduction of CD226 on immune cells in tumor-bearing hosts and prevalence of other axis members, raises several questions. Most notably, how much CD226 expression is necessary to drive functional responses following blockade of inhibitory receptors in the axis, and is this something that needs to be monitored to predict responses? As clinical efforts progress, it will be of interest to interrogate the relevance of CD226 dynamics in therapeutic responses.

### Functional and Therapeutic Implications

The importance of CD226 in shaping the overall immune response has been thoroughly described in the context of autoimmunity, cancer, and viral infections (48). CD226 orchestrates the net activity of innate (NK cells) and adaptive (T cells) immunity *via* interplay with CD155 and CD112 (8, 49). For example, high expression of cell-surface CD155 coupled with low human leukocyte antigen (HLA) expression increases the susceptibility of immature DCs to CD226-mediated killing by NK cells. This process bridges both innate and adaptive immunity by removing Th2-polarizing iDCs, thus skewing T helper cell polarization (50–52). CD226 has been shown to play a critical role in promoting broad T cell expansion, CD8^+^ T cell antitumor activity, and “adaptive” NK cell responses (37, 53–55).

A significant amount of information regarding the contribution of CD226 to immune responses has been gleaned from genetic- and biologics-based approaches in mice. In contrast to delayed tumor progression in TIGIT^-/-^ and CD96^-/-^, CD226-deficient mice exhibit increased susceptibility to MCA-induced fibrosarcomas as well as metastatic lung colonization (e.g., LLC lung and RM-1 prostate tumors) (19, 20, 56–58). Moreover, genetic ablation or antibody-mediated blockade of CD226 has been shown to abrogate the antitumor activity observed in CD96-deficient mice and in mice treated with anti-TIGIT, anti-PD-(L)1, and/or GITR (19, 20, 59, 60). Tumors propagated in CD226-deficient mice also exhibit increased expression of CD155 and CD112, further highlighting the dynamics within the CD226 axis (54, 57).

Given its critical immunostimulatory role, agonist approaches for CD226 seem attractive as a means to generate antitumor responses. However, CD226 is also expressed on platelets and has been associated with their adhesion/activation, potentially complicating the desired pharmacology profile (18, 61). Thus far, only one agent for CD226, LY3435151 (anti-CD226 agonist antibody, Eli Lilly), has progressed to clinical testing (NCT04099277, **Table 3**). Nevertheless, shortly after initiation, the phase 1 study for LY3435151 was terminated. Although one can speculate as to the reason(s) for study termination, given the critical position of CD226 in CD155 and CD112 axes, it will be of great interest to understand the limitations surrounding CD226 as a target for cancer immunotherapy.

**Table 3:** Ongoing or discontinued (terminated) clinical trials evaluating CD226 axis-related antibody-based therapies in cancer patients.

Target	Agent	Isotype	Phase	Indication	Study	Details
TIGIT	Tiragolumab (Roche)	hIgG1	3	NSCLC	NCT04294810 (SKYSCRAPER-01)	• PD-L1-selected • + atezolizumab
…	…	…	3	ES-SCLC	NCT04256421 NCT04665856 (SKYSCRAPER-02/C)	• + atezolizumab and chemo
…	…	…	3	NSCLC	NCT04513925 (SKYSCRAPER-03)	• + atezolizumab • + durvalumab • No progression on CRT
…	…	…	3	LA Esophageal	NCT04543617 (SKYSCRAPER-07)	• + atezolizumab
…	…	…	3	1L LA Esophageal	NCT04540211 (SKYSCRAPER-08)	• + atezolizumab and chemo
…	…	…	2	Cervical	NCT04300647 (SKYSCRAPER-04)	• PD-L1+ patients • + atezolizumab
…	…	…	2	NSCLC	NCT03563716 (CITYSCAPE)	• Chemo-naïve • + atezolizumab
…	…	…	2	NSCLC	NCT05034055 (SKYROCKET)	• SBRT • + atezolizumab
…	…	…	2	HNSCC	NCT04665843 (SKYSCRAPER-09)	• 1L PD-L1+ • + atezolizumab
…	…	…	2	Non-squam. NSCLC	NCT04958811	• + bevacizumab • + atezolizumab
…	…	…	2	SCLC	NCT04308785	• + atezolizumab • No progression on CRT
…	…	…	2	Melanoma	NCT05060003	• ctDNA+ following resection • + atezolizumab
…	…	…	2	Non-squam. NSCLC	NCT04619797 (SKYSCRAPER-06)	• + atezolizumab and chemo
…	…	…	2	Rectal	NCT05009069	• + atezolizumab following neoadjuvant chemo
…	…	…	2	HER2-G/GEJ	NCT04933227	• + atezolizumab and chemo
…	…	…	2	NSCLC	NCT04832854	• + atezolizumab • +/- neoadjuvant chemo
…	…	…	2	Melanoma (stage III)	NCT03554083 (NeoACTIVE)	• + atezolizumab and chemo
…	…	…	2	HNSCC	NCT03708224	• + neoadjuvant atezolizumab • + tocilizumab
…	…	…	2	Mixed	NCT04632992 (MyTACTIC)	• Platform study
…	…	…	1b/2	GEJC	NCT05251948 (Morpheus-C-Gastric)	• Platform study
…	…	…	1b/2	HCC	NCT04524871 (Morpheus-Liver)	• Platform study
…	…	…	1b/2	mUC	NCT03869190 (Morpheus-UC)	• Platform study • Post-platinum fail
…	…	…	1b/2	mPDAC	NCT03193190 (Morpheus-Pancreatic)	• Platform study • 1L/2L cohorts
…	…	…	1b/2	G/GEJ	NCT03281369 (Morpheus)	• Platform study • 1L/2L cohorts
…	…	…	1b	TNBC	NCT04584112	• + atezolizumab and chemo
…	…	…	1	MM/NHL	NCT04045028	• R/R setting • + daratumumab and rituximab
…	…	…	1	Mixed	NCT02794571	• Dose-escalation • + atezolizumab and chemo
TIGIT	Vibostolimab (Merck US)	hIgG1	3	NSCLC	NCT04738487	• PD-L1+ • + pembrolizumab and chemo
…	…	…	2	NSCLC	NCT04165070 (Keynote-01A)	• Treatment-naïve • + pembrolizumab and chemo
…	…	…	2	R/R heme malignancies	NCT05005442	• pembrolizumab co-formulation
…	…	…	2	NSCLC	NCT04725188	• Progression post-chemo/PD-1 • pembrolizumab co-formulation + chemo
…	…	…	2	Mixed	NCT05007106	• pembrolizumab co-formulation +/chemo
…	…	…	2	NSCLC	NCT04165070 (KEYMAKER-U01)	• + pembrolizumab and chemo
…	…	…	1/2	Melanoma	NCT04305041 (KEYMAKER-U02A)	• Platform study • PD-1 refractory • + pembrolizumab
…	…	…	1/2	Castration-resistant prostate	NCT02861573 (KEYNOTE-365)	• pembrolizumab co-formulation
…	…	…	1/2	Melanoma	NCT04305054 (KEYMAKER-U02B)	• Platform study • 1L patients • + pembrolizumab
…	…	…	1/2	Melanoma	NCT04303169 (KEYMAKER-U02C)	• Platform study • Neoadjuvant Tx • + pembrolizumab
…	…	…	1	Mixed	NCT02964013 (KEYNOTE-01A)	• Dose-escalation • + pembrolizumab and chemo
TIGIT	Ociperlimab (Beigene)	hIgG1	3	NSCLC	NCT04746924	• + tislelizumab
…	…	…	3	LA NSCLC	NCT04866017	• + tislelizumab vs durvalumab • + CRT
…	…	…	2	HCC	NCT04948697	• + tislelizumab and BAT1706
…	…	…	2	NSCLC	NCT05014815	• + tislelizumab and chemo
…	…	…	2	NSCLC	NCT04952597	• + tislelizumab and CRT
…	…	…	2	Esophageal	NCT04732494	• + tislelizumab
…	…	…	2	BTC	NCT05019677	• 1L • + tislelizumab and chemo
…	…	…	2	Cervical	NCT04693234	• + tislelizumab
…	…	…	2	BTC	NCT05023109	• Unresectable • + tislelizumab and chemo
…	…	…	1	Mixed	NCT04047862	• + tislelizumab
TIGIT	Domvanalimab AB154 (Arcus)	hgG1, FcγR-null*	3	NSCLC	NCT04736173	• PD-L1+ patients • 1L setting • + zimberelimab and etrumadenant
…	…	…	2	NSCLC	NCT04791839	• Prior checkpoint blockade • + zimberelimab and AB928
…	…	…	2	NSCLC	NCT04262856 (ARC-7)	• PD-L1+ patients • 1L setting • + zimberelimab and AB928
…	…	…	2	R/R melanoma	NCT05130177	• + zimberelimab
…	…	…	1	Mixed	NCT03628677	• Dose-escalation • + zimberelimab
TIGIT	BMS-986207 (BMS)	hgG1, FcγR-null*	2	NSCLC (stage IV)	NCT05005273	• + nivolumab and ipilimumab
…	…	…	1/2	Mixed	NCT04570839	• Dose-escalation • + nivolumab and COM701
…	…	…	1/2	Mixed	NCT02913313	• Dose-escalation • + nivolumab • + nivolumab and ipilimumab
…	…	…	1/2	MM	NCT04150965	• R/R setting • + chemotherapy
TIGIT	IBI939 (Innovent)	hIgG1*	1	Advanced lung cancer	NCT04672356	• Dose-escalation • + sintilimab
…	…	…	1	NSCLC	NCT04672369	• Dose-escalation • + sintilimab
…	…	…	1	Mixed	NCT04353830	• Dose-escalation • + sintilimab
TIGIT	Etigilimab (Oncomed)	hIgG1	2	Ovarian/ fallopian	NCT05026606	• + nivolumab
…	…	…	1/2	Mixed	NCT04761198	• + nivolumab
…	…	…	Term.	Mixed	NCT03119428 (terminated)	• Dose-escalation • + nivolumab
TIGIT	ASP8374^&^ (Astellas)	hIgG4	1b	Mixed	NCT03260322	• Dose-escalation • + pembrolizumab
…	…	…	1	Mixed	NCT03945253	• Japanese patients
…	…	…	1	Glioma	NCT04826393	• + cemiplimab
TIGIT	EOS884448 (GSK/iTeos)	hIgG1	1/2	Mixed	NCT04335253 (IO-002)	• Dose-escalation
…	…	…	1/2	Mixed	NCT05060432 (TIG-006)	• + pembrolizumab • + inupadenant
TIGIT	SGN-TGT (Seattle Genetics)	hIgG1 FcγR-enhanced	1	Mixed	NCT04254107	• Dose-escalation • Solid tumors and lymphomas + sasanlimab
TIGIT	COM902 (Compugen)	hIgG4*	1	Mixed	NCT04354246	• Dose-escalation
TIGIT	M6223 (Merck KGaA)	hIgG1*	1	Mixed	NCT04457778	• Dose-escalation • + bintrafusp alfa
TIGIT	AB308 (Arcus)	hIgG1	1	Mixed	NCT04772989	• Dose-escalation • + zimberelimab
TIGIT	BAT6021 (Bio-Thera)	hIgG1 FcγR-enhanced*	1	Mixed	NCT05073484	• Dose-escalation • + BAT1308
TIGIT	JS006 (Junshi Bio)	hIgG4	1	Mixed	NCT05061628	• Dose-escalation • + toripalimab
TIGIT	AK127 (Akesobio)	?	1	Mixed	NCT05021120	• Dose-escalation • + AK104
TIGIT x PD-1	AZD2936 (AstraZeneca)	?	1/2	NSCLC	NCT04995523 (ARTEMIDE-01)	• Bi-specific based on COM902
TIGIT x ?	AGEN1777 (Agenus/BMS)	?	1	Mixed	NCT05025085	• Bi-specific • Dose-escalation • + PD-1
PVRIG	COM701 (Compugen)	hIgG4	1/2	Mixed	NCT04570839	• + nivolumab and BMS-986207
…	…	…	1	Mixed	NCT03667716	• Dose-escalation • + nivolumab
…	…	…	1	Mixed	NCT04354246	• Dose-escalation • + COM902
PVRIG	GSK4381562 (Compugen)	hIgG1	1	Mixed	NCT05277051	• + Dose-escalation • + dostarlimab
CD226	LY-3435151 (Eli Lilly)	Unknown	Term.	Mixed	NCT04099277 (terminated)	• Dose-escalation • + pembrolizumab
CD96	GSK6097608 (GSK/23andMe)	hIgG1	1	Mixed	NCT04446351	• Dose-escalation • + dostarlimab

*to be confirmed; ^&^discontinued; LA, locally advanced; ES-SCLC, extensive-stage small cell lung cancer; mUC, metastatic urothelial carcinoma; mPDAC, metastatic pancreatic ductal adenocarcinoma; G/GEJ, gastric or gastroesophageal junction adenocarcinoma; GEJC, gastroesophageal junction carcinoma, TNBC, triple-negative breast cancer; R/R, relapsed/refractory; MM/NHL, multiple myeloma/non-Hodgkin lymphoma; BTC, biliary tract carcinoma; FTIH, first time in human study; CRT, chemoradiotherapy. The “?” symbol in Table 3 symbolizes “undetermined”.

## Key Ligands in the Axis: CD155 and CD112

### Discovery, Structure, and Interactions

Identified several decades before an association with relevant immune receptors, CD155 (poliovirus receptor [PVR], necl-5) and CD112 (PVRL2, nectin-2) represent structurally similar immunoglobulin superfamily (IgSF) adhesion glycoproteins (62–64). There are several isoforms of CD155 (α, β, γ, δ) and CD112 (short, CD112α; long, CD112δ), each with slightly different structural characteristics and tissue distribution (8, 65, 66). While the α and δ isoforms of CD155 code for cell-surface expressed CD155, the β and γ isoforms lack transmembrane (TM) domains, resulting in a soluble/secreted proteins that are not present in rodents (7, 65, 67). The extracellular region of CD155 and CD112 is comprised of an N-terminal variable (V) domain and constant (C1-C2) domains (24, 68–70). Like CD226, the V domain contains conserved ‘lock’ (AX_6_G) and ‘key’ (T[F/Y]P) motifs that are critical for mediating homo- and heterophilic interactions (24, 25, 68). Appropriately, CD155 and CD112 are capable of *trans* interactions, which can facilitate sustained cell-to-cell contact and immunoregulation (71, 72). Contrary to many nectin proteins, CD155 fails to exhibit strong homophilic interactions (71, 73). Similar evidence suggests that CD112 functions as a monomer, but also exhibits proclivity for homodimerization (74).

Outside of their conserved binding motifs, CD155 and CD112 exhibit nuances in binding orientation depending on the partner. For example, both CD155 and CD112 have been suggested to form heterotetrameric structures with TIGIT, where TIGIT dimers are sandwiched between CD155 or CD112 monomers; while other CD155 (e.g., CD226 and CD96) and CD112 complexes (e.g., CD226) are thought to be more conventional monomeric interactions (**Figure 1**) (25, 68–70). Differential structural attributes of TIGIT, CD96, and CD226 provide a partial explanation for their relative affinity to cell surface CD155 (26, 75). However, this is likely more complex given the variegated glycosylation patterns of the axis proteins. As mentioned previously, this affinity gradient, which favors inhibitory receptor binding (i.e., TIGIT and CD96), is reminiscent of the archetype CTLA-4 axis, whereby CTLA-4 effectively outcompetes costimulatory receptor CD28 from binding to B7 (CD80/86) (5). In a similar fashion, the inhibitory receptor PVRIG demonstrates a greater affinity for CD112 relative to CD226 (9).

### Expression and Regulation

Despite detectable levels on multiple cell types (e.g., myeloid and epithelial cells) under physiological conditions, CD155 and CD112 are often elevated in various solid and hematological malignancies, correlating with a worse overall survival (12, 76–83). These correlations are not ubiquitous, however, as lack of expression in hepatocellular carcinoma (HCC) has been shown to be prognostically unfavorable (84, 85). Indeed, akin to PD-L1, CD155 expression has also been shown to be predictive of response to ICI (e.g., anti-PD-1 and anti-PD-1/CTLA-4) (86). This underscores the need for a clear etiological understanding and cancer-immune interplay in each indication. Moreover, treatment status must be considered, as CD155 and CD112 can be induced downstream of a range of cellular insults or stimuli known to activate the DNA damage response (DDR) pathway (87–92).

In addition to relatively broad expression by human tumor tissue and stromal populations, CD155 and CD112 are expressed on myeloid cells, such as monocytes and various subsets of dendritic cells (DCs) (50, 93). Given the reported expression of CD155 on follicular DCs, a key component of B cell follicles/germinal centers (GC), the involvement of the CD226 axis in tertiary lymphoid structure (TLS) biology may warrant further exploration (93, 94). This is particularly attractive in light of recent correlations between TLS generation/presence and response to cancer immunotherapy (95–97). One area where CD155 and CD112 appear to diverge is in lymphocyte expression, whereby CD155 can be induced on highly activated T cells (87, 98, 99). While the functional consequences of CD155 on activated T cells remains to be determined, CD155 has been associated with thymic selection *via* lymphocyte retention, underscoring its adhesion properties and potential impact on T cells (100). The expression of CD155 on T cells may also introduce complexity into the mechanistic interpretation and be derivative of certain experimental models. For example, CD155 induction on T cells may complicate our understanding of how anti-CD96 antibodies mediate functional activity even under conditions of T cell isolation (i.e., agonist activity or blockade of CD96:CD155 T cell-to-T cell interactions).

As the name suggests, CD155 or poliovirus receptor (PVR) serves as a point of cellular entry for poliovirus and, similar to the described role for soluble intercellular adhesion molecule-1 (ICAM-1) in response to rhinovirus infection, soluble CD155 has been proposed to be a partial serum-based sink for poliovirus (65, 101). Interestingly, soluble CD155 and CD112, have also been described in the serum of cancer patients and are often correlated with disease stage (7, 66, 102, 103). However, the specific contribution of soluble CD155 and CD112 to disease progression remains to be determined. Despite the longstanding awareness of CD155 and CD112 expression in cancer, an understanding of their contribution to the regulation of immune function and migration was lacking until a connection with the CD226 was established (8–10, 70, 104, 105). Functional characteristics and therapeutic implications for each interaction will be discussed in the following sections.

## TIGIT

### Discovery, Interactions and Structure

Several years after the description of CD226 and its association with CD155, T cell immunoglobulin and ITIM domain (TIGIT; V-set and transmembrane domain-containing 3 [Vstm3]; V-set and Ig domain-containing 9 [Vsig9]; Washington University Cell Adhesion Molecule [WUCAM]) emerged as an important member of the CD226 axis (10, 23, 93). The discovery of TIGIT was aided by searching for predicted structural similarities with cell-surface immune receptors, like PD-1. After its initial identification as a receptor for CD155, an additional ligand (CD112) and a role for TIGIT in NK cell modulation was described (10).

The extracellular region of TIGIT is comprised of a single IgV, and, consistent with other receptor-ligand pairs in the axis, *trans* interactions are facilitated by a lock-and-key motif in the N-terminal domain (68) (**Figure 2**). The majority of TIGIT function is tied to CD155, with binding to CD112 representing a lower affinity interaction (~30-fold *via* surface plasmon resonance [SPR]) (106). TIGIT has also been shown to interact with CD113 (PVRL3) and nectin-4 (PVRL4); however, clear functional implications remain to be seen (23, 107). Primarily facilitated by isoleucine (Ile) 42 (human; an analogous residue exists in mice) and the associated parallel interface, TIGIT exhibits a propensity for dimerization on the surface of cells (68). Accordingly, *cis* multimeric interactions with CD226 have also been reported, providing an additional means of CD226 regulation (36, 60).

The intracellular domain of TIGIT harbors an immunoreceptor tyrosine tail (ITT)-like phosphorylation motif and a conserved ITIM (LSYRSL) (106). While the role of each motif has been evaluated in mice and man, the relative contribution of ITT/ITIM to cell intrinsic TIGIT activity appears to be more contextual in humans (**Table 2**). Using a modified cytotoxicity system (YTS and 721.221 cells), Stanietsky et al. demonstrated that rescue of murine CD155-mediated NK cell suppression required mutation of both ITT and ITIM tyrosine residues (108). The same authors demonstrated that site-directed mutagenesis of only the ITIM (Y221) in human cells was able to abrogate CD155- and CD112-induced suppression of NK cell cytolytic activity (10). Further, a separate set of studies ascribed the ITT as the critical moiety for TIGIT-mediated inhibition of human NK cell IFNγ production, granule polarization, cellular cytotoxicity (34, 35). Recently, Banta and colleagues demonstrated that the intracellular domain of TIGIT was largely dispensable for impairment of CD226 phosphorylation (36). Rather, as previously mentioned, *cis* multimeric interactions with CD226 and/or ligand competition were found to drive TIGIT inhibition of CD226 co-stimulation. While this work brings into question the relative contribution of intrinsic signaling to TIGIT function (**Table 2**), it will be necessary to understand if it is selective to the experimental system, specific to TIGIT/CD226, or a more ubiquitous phenomenon.

Apart from ligand sequestration, TIGIT has been credited with additional mechanisms of action, which may be extrinsic or intrinsic depending on cell type. For instance, Yu et al. utilized human T cell and monocyte-derived DC co-cultures to demonstrate that unabated TIGIT : CD155 ligation alters antigen presenting cell (APC) cytokine profiles, indirectly impairing T cell responses (23). While multiple studies have revealed similar cell-extrinsic function of TIGIT, T and NK cell-intrinsic TIGIT signaling has also been described in both mice and man (22, 58, 108–112). For example, Joller et al. utilized an anti-mouse agonistic TIGIT antibody, in concert with TIGIT^-/-^ mice, to characterize intrinsic TIGIT signaling in CD4^+^ T cells (111). The authors found that components of the T cell receptor complex (e.g., TCRα and CD3ε) were directly modulated following TIGIT engagement, adding another layer of immunoregulation to the mechanistic story of TIGIT.

### Expression and Regulation

In the peripheral compartment, TIGIT is broadly represented on T and NK cell subsets, with noteworthy representation on regulatory T (Treg) cells, NKT cells, T follicular helper (Tfh) cells, and γδ T cells (**Table 1**) (11, 13). Apart from prominent expression of TIGIT on Treg cells, expression patterns for CD226 axis members ostensibly diverge when it comes to memory T cell populations (12). TIGIT and PVRIG are elevated on human terminally differentiated CD45RA+ TEMRA cells, whereas CD96 expression is mainly restricted to central memory (CM)/effector memory (EM) T cells (113, 114). It is intriguing to speculate about the functional effects that blockade of each immune receptor may have on peripheral or tumor/tissue-resident memory T cells. However, additional characterization, particularly on tumor-infiltrating immune populations, is required to determine if these expression patterns are indicative of any meaningful functional differentiation.

Consistent with its relatively high level of expression, TIGIT has been ascribed a role in the homeostasis and function of Treg cells. Indeed, TIGIT^+^ Treg cells have been shown to be highly immunosuppressive relative to their TIGIT^-^ counterparts, inhibiting T helper (Th)1 and Th17 responses *via* induction of fibrinogen-like 2 (Fgl2) (59, 113, 115). This observation may be particularly relevant in cancer due to the elevated expression of TIGIT on intratumoral Treg cells (58, 116). As such, cellular depletion represents a plausible therapeutic mechanism for Fc-enabled TIGIT antibodies in cancer patients (discussed later).

In addition to readily detectable baseline expression, TIGIT is also upregulated on T and NK cells following activation. As exhausted T cells (T_EX_) are largely a product of tonic TCR stimulation, TIGIT has become a mainstay in several T cell ‘exhaustion signatures’ and has been implicated as a potential node for functional reversion of T_EX_ (117, 118). However, because T cell exhaustion is a complicated process that is encumbered by progressive stages of epigenetic modification, TIGIT blockade alone is likely insufficient for meaningful functional rescue of fully exhausted T cells (119). Perhaps one way to determine a functional role for TIGIT in this process is to longitudinally characterize the impact of TIGIT inhibition on the arc of T cell activation to terminal exhaustion. Adapting these observations to the TME may inform whether TIGIT blockade could effectively prevent exhaustion or simply accelerate it. Alternatively, one could consider TIGIT blockade in concert with epigenetic modulators as a potential strategy to unmask and concomitantly enhance the activity of T_EX_ (120). Regardless of potential functional implications, robust expression on T_EX_ is consistent with progressive TIGIT induction following immune activation.

TIGIT expression is detectable in a wide range of human cancers and is often tightly correlated with T cell transcripts (CD4 and CD8A) (11). While its expression profile is largely consistent between the periphery and TME, TIGIT is upregulated on various TIL populations and is often accompanied by the expression of other activation-induce immune receptors, such as CD244 (2B4), TIM-3, and PD-1 (121, 122). Similar to the expression of TIGIT on TEMRA cells in the periphery, terminally differentiated intratumoral CD8+ T cells also express elevated levels of TIGIT. One could hypothesize that these cells are derivative of TIGIT^low^ stem-like T cells residing within defined TME niches or in the periphery. Alternately, these could be newly infiltrating cytotoxic T cells that progressively acquire TIGIT during *in situ* differentiation or repeated TCR activation (123, 124).

### Functional and Therapeutic Implications

No baseline developmental or immunological defects have been described in TIGIT (VSTM3)-deficient mice (C57BL/6 background) (125). However, the severity of induced autoimmune manifestations, such as myelin oligodendrocyte glycoprotein (MOG)-dependent encephalomyelitis (EAE) or graft-vs-host disease (GVHD) is increased relative to wildtype mice. In the context of cancer, TIGIT deficiency, or prophylactic antibody blockade, yields a modest level of protection against primary tumor growth in mice (19, 126–128). By contrast, TIGIT monotherapy is often ineffective in mice with established lesions (60). Therefore, combination with other ICI, such as anti-PD-(L)1, has been utilized to achieve more pronounced antitumor responses in a range of tumor models (60, 129, 130). In addition, the antitumor activity of murine anti-TIGIT antibodies has been tied to Fc-Fcγ receptor co-engagement (15, 131). This dependency is noteworthy given the myriad of anti-TIGIT isotypes currently under clinical evaluation (discussed later).

While the focus has largely been on solid tumors, a role for TIGIT in hematological malignancies has also been described. TIGIT is most notably upregulated on CD8+ T cells in multiple myeloma (MM), in both mice and man (128, 132, 133). Akin to what has been suggested in solid tumors, repeat antigen exposure and associated progressive exhaustion have been implicated in the upregulation of TIGIT in this setting and is of particular interest in the context of relapsed/refractory (R/R) disease. Accordingly, inhibition of TIGIT (genetic- or antibody-based) results in T cell-dependent antitumor responses in several syngeneic models of MM and is currently being evaluated in clinical studies (e.g., NCT04045028 and NCT04150965) (128). TIGIT has also been implicated in other heme cancers, such as follicular lymphoma (FL) and acute myeloid leukemia (AML) (134–136).

## CD96

### Discovery, Interactions and Structure

CD96 (T-cell activation, increased late expression [TACTILE]) was first identified as an orphan receptor on AML and T-cell acute lymphoblastic leukemia (T-ALL) cell lines (137). A functional role for CD96 wasn’t identified until half a decade later when the interaction between CD96 on NK cells and CD155 on tumor cells was described (105). Interestingly, this finding was influenced by observations involving NK cell expressed CD226 (8): The authors noted that, while the human NK cell line NK92 bound to the extracellular domain of CD155, the cells lacked detectable expression of CD226, thereby implicating a similarly structured receptor, CD96. This finding introduced a possible functional role for CD96 on immune cells and, with CD226, provided a framework for a novel immunoregulatory axis.

CD96 is comprised of three Ig-like extracellular domains and a flexible membrane-proximal stalk region containing multiple *O*-linked glycosylation sites (138) (**Figure 2**). Only the N-terminal domain (D1) contains a lock-and-key motif critical for binding CD155. In addition to CD155 binding, CD96 has also recently been shown to interact with human CD111; however, a functional role for this interaction remains to be elucidated (70, 75). While much of the biophysical data generated suggest that CD96 functions as a monomer, the possibility of CD96 oligomerization (i.e., *cis* interactions) needs to be evaluated in more complex systems to better understand how CD96 behaves on cells. As an example, the anti-mouse CD96 clone 8B10 (D2 binder) exhibits partial tumor control in an experimental model of metastases, yet is not entirely dependent on the presence of CD155 (139). This leads one to question if *cis* interference has a contextual biological role for CD96, if there are other important *trans* interactions, or whether this is the result of technical limitations.

Three isoforms of CD96 have been identified in humans, with two membrane-tethered isoforms (a longer variant [1] and a shorter variant [2]) and a less studied soluble isoform (variant 3) (75, 138, 140). Due to a truncated exon 4, the V2 isoform lacks a stretch of amino acids (~18) in the second Ig domain, resulting in an abbreviated loop structure. While less is known about the soluble form (V3), the V2 isoform is reported to be the most widely expressed and exhibits the highest affinity for CD155. The first domain of CD96 is reported to contain the epitope(s) required for CD155 binding while the second domain supports the magnitude/strength of binding (138). The intracellular domain of CD96 contains multiple tyrosine residues, with a prototypical inhibitory motif (ITIM, IXYXXI) that is conserved across species (138). The presence of an ITIM, coupled with the capacity for direct cross-competition with CD226/CD155 binding, suggests that CD96 functions as an inhibitory receptor. However, the categorization of CD96 as an inhibitory receptor has been mired due to (i) limited knowledge of CD96 signaling, (ii) conflicting functional data (particularly with NK cells), and (iii) the existence of a YXXM motif (YHEM) in primate CD96 (**Figure 1**) (105). Recently, Chiang et al. ascribed costimulatory properties to both mouse and human CD96 (141). While certainly not the only description of CD96 as a costimulatory receptor, caution should be taken regarding the interpretation of data in certain experimental systems. Because CD155 can be induced on TCR-activated T cells and the primordial involvement of nectin/necl proteins in cell adhesion, it is important to understand if the functional effects elicited by anti-CD96 are simply the result of blocking CD155-CD96 t*rans* interactions (87, 98, 99). In addition, while several costimulatory receptors (e.g., inducible T-cell co-stimulator [ICOS]) harbor a YXXM motif, defining anti-CD96 functional directionality based on its presence may be shortsighted, as CD96 lacks a YxxM in mice and inhibitory receptors like CTLA-4 also contain similar sequences without a clearly defined functional role (142, 143).

### Expression and Regulation

CD96 is expressed at baseline by several T cell populations (αβ and γδ), NK/NKT cells, and select B cell subsets in both mice and man (**Table 1**). The expression of CD96 on primary human immune cells is most evident on CD56+ NK cells and CD8+ T cells, with prominent representation on central and effector memory T cells (11, 113). Interestingly, human bone marrow-resident lymphoid tissue (lt) NK cells, which exhibit an overlapping transcriptional profile with tissue-resident memory CD8+ T cells, express both CD96 and TIGIT (144). Consistent with its alias (T-cell activation, increased late expression or ‘TACTILE’), CD96 expression also increases following TCR- or cytokine-based activation (e.g., interleukin [IL]-18 for T cells and TGF-β for NK cells) (39, 145, 146). In a recent study, Lepletier et al. described a near-homogenous level of CD96 and CD226 co-expression on TCR-activated peripheral human CD8+ T cells (11).

CD96 is also highly represented on rodent and human T cells and NK cells within the TME. A marked correlation between CD96/TIGIT messenger(m) RNA levels and CD3E/CD8a/CD4, with similar observations at the protein level (i.e., T cells), can be seen across multiple tumor types (11). Relative to T cells, the correlation between CD96 and NK cell-related genes is more infrequent despite being strong for specific indications such as HCC, head and neck squamous cell carcinoma (HNSCC), stomach adenocarcinoma, and melanoma (11, 147). Relative expression of CD96 and TIGIT on HCC-derived NK cells was shown to be dependent on tissue sub-localization, with TIGIT evenly represented across NK cells in the normal liver and intra/peritumoral space and CD96 more restricted to intratumoral NK cells (39, 148). CD96 expression in HCC is inversely correlated with several functional markers of NK cells, including T-bet (TBX21), perforin (PRF1), and granzyme B (GZMB) (39). Interestingly, TGF-β1 has been implicated in the induction of CD96 and associated CD226 downregulation in HCC, establishing a connection between two immunosuppressive pathways (39). More recently, CD96 was found to be co-expressed with PD-1 on TCF1+ exhausted precursor T cells in cervical tumors, a characteristic that the authors tied to therapeutic insensitivity (149). CD96 expression has also been noted in various hematological malignancies, such as T-ALL, myelodysplastic syndrome (MDS), and leukemic stem cells (LSCs) in AML (137, 150, 151). However, the biological relevance of CD96 in heme malignancies and its potential as a therapeutic target remains to be determined.

### Functional and Therapeutic Implications

Similar to TIGIT, CD96-deficient mice (C57BL/6 background) do not exhibit overt baseline immunological defects (19, 152). However, challenge of CD96^-/-^ mice with lipopolysaccharide (LPS) results in hyperinflammation characterized by enhanced IFNγ production by NK cells. Augmented NK cell function in CD96^-/-^ mice is best exemplified by improved antimetastatic activity in B16F10 melanoma, RM-1 prostate cancer, and EO771 breast cancer models (127). Similar CD8+ T cell-dependent effects have been observed in CD96-defificent mice, albeit in the context of concomitant PD-1 (*Pdcd1*) knockout (114, 152). Notably, inflammatory responses and related antitumor activity in CD96^-/-^ mice are abrogated following genetic ablation of CD226, underscoring the level of axis interplay (19, 152). Similar improvements in antitumor responses have been observed in mice following therapeutic inhibition of CD96, alone or in combination with other immune checkpoint inhibitors (114, 149). As an example, following neoadjuvant anti-PD-1 and gemcitabine, adjuvant therapy with anti-CD96 resulted in a significant survival benefit in pancreatic ductal adenocarcinoma (PDAC) tumor-bearing mice (153). Improvement in overall survival in this model was not solely driven by attenuation of primary tumor growth, but also by NK cell-dependent control of metastatic spread (153). Combination benefit of CD96 and PD-1 blockade has also been shown in other immunotherapy-insensitive models, such as the TC-1 (HPV+) tumor model (149).

Despite the wealth of information supporting an inhibitory role for CD96 in mice, functional data in humans is largely absent. Therefore, the progression of clinical-stage anti-CD96 antibodies, such as GSK6097608 will add important data to better understand mechanism of CD226 axis dynamics.

## PVRIG

### Discovery, Interactions and Structure

PVR-related Ig domain-containing (PVRIG) is the most recently discovered CD226 axis member (9). While much less is known about PVRIG, the sequestration of CD112 away from CD226 represents a regulatory mechanism similar to that utilized by TIGIT and CD96 co-inhibitory receptors (**Figure 1**) (9, 12). Alongside this potential for ligand competition, PVRIG has the capacity for intrinsic inhibitory signaling – which is likely facilitated by an ITIM-like motif found in the intracellular domain of PVRIG (9) (**Table 2**). Interestingly, this inhibitory domain is lacking in mice, suggesting that PVRIG function in mice is more dependent on ligand competition or is tied to an alternative mechanism that has yet to be described (**Figure 2**).

### Expression and Regulation

Like TIGIT and CD96, PVRIG is expressed on various T and NK cell subsets and is upregulated on T cells following TCR stimulation (9). Elevated PVRIG expression has been described on exhausted T cells in human tumors, with noteworthy expression on TILs from ovarian, kidney, lung, endometrial, and breast cancers (12, 154). Similarly, antigen specific CD8+ T cells from virally infected mice exhibit similar upregulation of PVRIG as cells transition from activation to exhaustion (155). Despite lack of induction on *in vitro*-activated NK cells, increased PVRIG expression is observed on tumor-infiltrating NK cells (12). To date, little is known about regulation of PVRIG expression on different immune cell subsets.

### Functional and Therapeutic Implications

Genetic- or biologics-based inhibition of PVRIG has been shown to impair tumor growth, particularly when combined with anti-PD-L1 (76, 155, 156). Notably, tumor-infiltrating CD8+ T cells and NK cells from PVRIG-deficient mice exhibit increased proinflammatory cytokine production, suggesting that PVRIG is involved in direct or indirect modulation of tumor-infiltrating T/NK cells (155, 156). In humans, *in vitro* blockade of PVRIG : CD112 binding results in enhanced TCR signaling and, when combined with trastuzumab (anti-HER2) and anti-TIGIT, potentiates ADCC activity and IFNγ production by NK cells (9, 157).

## Clinical Landscape

### Overview

This current clinical landscape for the CD226 axis is heavily represented by TIGIT antibodies, with an increasing number of agents at various stages of clinical evaluation (158, 159). Two PVRIG antagonist antibodies (COM-701, Compugen; GSK4381562, GlaxoSmithKline [GSK]), a single CD96 antibody (GSK6097608; GSK/23andMe), and a recently discontinued CD226 agonist antibody (LY-3435151, Eli Lilly & Co) round out the rest of the agents under clinical testing (**Table 3**).

### TIGIT

As peculiar as it may sound, given that several agents have progressed into late-stage studies, the clinical landscape for TIGIT is just starting to take shape. Recent positive signs from CITYSCAPE (NCT03563716), a first-line NSCLC (1L) phase 2 clinical trial exploring atezolizumab (anti-PD-L1)/tiragolumab (anti-TIGIT) versus atezolizumab/placebo, were on display at American Society of Clinical Oncology (ASCO) 2020 (160). The most striking observation was the large jump in overall response rate (ORR) in PD-L1^high^ patients (PD-L1 TPS > 50%; N=29 patients in each group) from 24% in the control arm (atezolizumab + placebo) to 66% with the combination. At first glance, this ORR compares favorably to similar ICI combinations (e.g., anti-PD-1/CTLA-4, Checkmate-227/NCT02477826) and is comparable to anti-PD-1 + chemotherapy (Keynote-189/NCT02578680) in a similar NSCLC patient subset (N=202 patients) (161). These data have recently enabled a breakthrough therapy designation by the FDA for 1L PD-L1^high^ NSCLC. However, given the relatively small number of patients in CITYSCAPE, phase 3 studies from Roche and others will be critical for determining the robustness of the combination. Moreover, despite these promising results, both atezolizumab (18% [N=39 patients]) and the combination (16% [N=38 patients]) failed to elicit meaningful responses in PD-L1^low^ patients (TPS 1-49%) relative to historical use of anti-PD-1 or anti-PD-L1 + chemotherapy (e.g., Keynote-407/NCT02775435 and Impower150/NCT02366143) (162, 163). This lack of overt activity across NSCLC patients may not be all that surprising given the nature of TIGIT antibodies (i.e., activated T cell-orientation and combination dependence), however as clinical studies progress, it will be important to establish a mechanistic understanding for the reduced activity of the combination in these patients.

The safety profile of tiragolumab combined with atezolizumab was similar to that of atezolizumab alone. Immune-related adverse events (IRAEs) were more frequent with the combination (69% versus 47%) but these were primarily manageable grade 1 and 2 immune-mediated AEs. There were also a similar number of grade 3+ AEs in the two groups (48% versus 44%) suggesting that this ICI combination will be better tolerated than ICI/chemo combinations (Keynote-189/NCT02578680, pembrolizumab + chemo [67.2%]) (161). With respect to AEs that demonstrated a >5% difference between arms, infusion-related reactions (IRR), pruritus, rash, were more frequent with the combination whereas dyspnea, productive cough, and hypercalcemia were more frequent with atezolizumab monotherapy.

Tiragolumab/atezolizumab combinations have since expanded into a suite of late-stage clinical trials dubbed the SKYSCRAPER trials. These trails are spread across indications, with SKYSCRAPER-01, -02, -03 and -06 in lung cancers, SKYSCRAPER-04 in PD-L1^+^ cervical cancer, and SKYSCRAPER-07, -08 and -09 in the ENT (esophageal or head and neck) sphere. Other indications including urothelial carcinoma, pancreatic cancer, and esophageal cancer are being explored *via* MORPHEUS umbrella studies (**Table 3**).

Other TIGIT molecules, including vibostolimab (MK-7684, Merck US) and ociperlimab (BGB-A1217, Beigene) are close behind with similar studies in NSCLC and a wave of studies in alternative indications (**Table 3**). Merck is also progressing vibostolimab and pembrolizumab as a co-formulation through phase 2 studies. This effort underscores the marriage between PD-1 or PD-L1 and TIGIT intervention strategies, however, time will tell if concurrent administration is sufficient or if dosing flexibility is required to enable optimal responses. Other agents, including Arcus (domvanalimab) and BMS (BMS-986207) are entering phase 2 and 3 studies. As opposed to the number of Fc-enabled TIGIT antibodies like tiragolumab and vibostolimab, agents like domvanalimab and BMS-986207 have attenuated Fc regions. Adding to this diversity, Fc-enhanced (SGN-TGT and BAT6021) as well as bi-specific (AZD2936 and AGEN1777) TIGIT antibodies have recently entered clinical development (**Table 3**). Given the non-clinical data suggesting the importance of Fc-FcγR co-engagement for anti-TIGIT function, the divergence in Fc biology between clinical molecules will be something to consider as studies read out.

Outside of solid tumor indications, TIGIT is also being evaluated in heme malignancies, such as MM and non-Hodgkin lymphoma (B-NHL) (**Table 3**). As discussed previously, TIGIT has been mechanistically linked to T cell exhaustion in MM and has shown promise as a therapeutic target in non-clinical models (128, 164). It will be interesting to see how well these findings translate to the clinical space, and if this is unique to TIGIT or if the therapeutic potential extends to other CD226 axis members.

### PVRIG and CD96

As of Q2 2022, COM701 (IgG4) and GSK4381562 (IgG1) are the only two PVRIG molecules under clinical evaluation. Notably, COM701 demonstrated some early signs of activity, with a preliminary single-agent disease control rate (DCR) of 69% (165). Compugen also initiated a phase 1/2 study evaluating the triple combination of COM701, anti-PD-1 (nivolumab), and BMS-96820 (anti-TIGIT) in ovarian, endometrial, and select PVRL2^high^ cancers. Clinical studies for CD96 are even more nascent, with a single molecule (GSK6097608, IgG1) in dose escalation as a monotherapy and in combination with anti-PD-1 (**Table 3**).

## Considerations for the CD226 Axis

### Stronger Together? Targeting Multiple CD226 Axis Members

Given the nuances in expression profiles and potential for promiscuity within the CD226 axis, it is intriguing to consider the possibility for compensatory regulation between different axis members, particularly under therapeutic pressure (**Figure 1** and **Table 1**). These characteristics also highlight the potential of therapeutic collaboration in order to prevent inhibitory exigencies and/or increase coverage of various immune subsets and regulatory nodes within the axis (12). Several lines of non-clinical evidence directly or indirectly support co-inhibition of CD226 axis members. For example, antibody-mediated blockade of PVRIG has been shown to both induce rapid receptor internalization and increase TIGIT expression on antigen-specific CD8+ T cells (12). Similar dynamics have been observed with the ligands in the axis (83, 166). Moreover, genetic- or biologics-based co-blockade of TIGIT and CD96 has been shown to improve tumor control while co-blockade of TIGIT and PVRIG has also been shown to promote NK cell function (152, 157).

### A PD-(L)1 Partnership

Although difficult to accurately assess due to the potential concomitant impact on TCR and CD28 activity, PD-1 signaling has been described to attenuate CD226 activity *via* SHP2-mediated dephosphorylation (20, 167) (**Figure 1**). This suggests that any CD226 signaling mediated by anti-TIGIT-, CD96- or PVRIG-based ligand redirection has the potential to be undercut by PD-(L)1 activity. Therefore, it is logical that CD226 axis therapeutics may need to be considered in the context of PD-(L)1 pathway blockade in order to reveal their full functional potential. Thus far, the available non-clinical data are consistent with this hypothesis. However, it will be interesting to see how the clinical space evolves, and whether or not PD-(L)1 blockade will become a prerequisite for the efficacy of all of the CD226 axis-based strategies.

### Potential Utility of Fc-FcγR Co-Engagement

Given the relative breadth of non-clinical studies, it is not surprising that much of the data describing a potential role for Fc biology in the efficacy of antibodies targeting CD226 axis members has been restricted to TIGIT (15, 16, 114). An underlying point of contention has been the potential for antibody-mediated cellular depletion, and whether this is beneficial or detrimental, either due to safety concerns or impairment of efficacy (60, 130, 168–170). TIGIT is highly expressed on both peripheral and tumor infiltrating Treg cells. While selective depletion of Treg cells in the TME could relieve a suppressive barrier and promote antitumor responses, depletion of Treg cells in other tissues could impair peripheral tolerance and result in autoimmune manifestations, as seen in patients treated with mogamulizumab, an afucosylated antibody targeting CCR4-expressingTreg cells (11, 58, 171, 172). One could postulate that TIGIT+ effector/cytotoxic T cells would also be a target for depletion, which may attenuate the desired therapeutic effect. Thus, if these concerns were overwhelming, pursuing an Fc-attenuated TIGIT antibody would appear logical. However, given that TIGIT expression is elevated on terminally exhausted T cells, one could also posit that depleting these cells in the TME would permit the establishment of more functional T cell populations, leading to a net beneficial effect. Multiple factors, including differential cellular thresholds for target opsonization, antibody affinity, presence of effector cells mediating depletion, and FcγR polymorphisms need to be considered (14, 173, 174). Moreover, while safety concerns have been allayed with the clinical progression of Fc-competent antibodies, various non-clinical studies have demonstrated a benefit and, in some cases, a requirement for intact Fc biology to facilitate anti-TIGIT function (15, 60, 114, 169). Although the exact mechanism(s) responsible for improved anti-TIGIT activity remains to be determined, it has been suggested that Fc-FcγR co-engagement drives myeloid activation and/or T cell-APC immune synapse quality (15, 131, 169). Clinical efficacy and safety data for recently developed Fc-enhanced TIGIT antibodies should also provide visibility on potential advantages or disadvantages associated with this format. Ultimately, the clinic will be the proving ground for the seemingly disparate Fc variants under evaluation; that is, to determine if pure antagonism, effector biology, or a mixture of mechanisms are required for optimal patient responses.

Limited Fc-based characterization has been conducted for rodent/human PVRIG or human CD96 antibodies (139). However, it is important to consider that the optimal therapeutic potential for each target must integrate a thorough understanding of Fc biology. Finally, given the breadth of TIGIT antibodies with distinct Fc regions currently under clinical evaluation (**Table 3**), broader mechanistic insights into anti-TIGIT biology and whether these characteristics extend to other members of the CD226 axis, may begin to surface in the coming years.

## Closing Remarks

In an effort to provide a framework for our evolving understanding of the CD226 axis in cancer, we discuss available non-clinical data and give an overview of the current clinical landscape. However, despite an understanding of the differential functional characteristics and expression profiles of each axis member, many questions remain regarding the mechanistic dynamics and contextual roles for each receptor. Some examples include (i) the functional implications of the variegated receptor expression (particularly with memory or stem-like memory T cell populations and regulatory T cells), (ii) the cell-intrinsic impact of individual receptor signaling, (iii) the functional consequence of ligand/receptor dynamics in different tissues (e.g., TME versus peripheral blood), (iv) the *trans*/*cis* interactions critical for activity, and (v) the contribution of FcγR biology to the function of antibodies for each receptor. As clinical programs advance and interest expands, some of these questions may begin to be addressed. Overall, it will be intriguing to see how therapeutic strategies for each receptor evolves and what mechanistic learnings precipitate from an increased amount of activity around the targets.

## Author Contributions

MC and JW generated the original draft manuscript. JW, KH, SY, and JS reviewed, edited, and added content. All authors contributed to the article and approved the submitted version.

## Conflict of Interest

All authors are employees of GlaxoSmithKline.

## Publisher’s Note

All claims expressed in this article are solely those of the authors and do not necessarily represent those of their affiliated organizations, or those of the publisher, the editors and the reviewers. Any product that may be evaluated in this article, or claim that may be made by its manufacturer, is not guaranteed or endorsed by the publisher.
